# Modulus of Elasticity and Flexural Behavior of Glulam Beams Reinforced with Steel Mesh in Different Mesh Openings

**DOI:** 10.3390/ma16124307

**Published:** 2023-06-10

**Authors:** Hilal Ulaşan, Agron Bajraktari, Nihat Döngel, Hasan Özgür Imirzi, Cevdet Söğütlü

**Affiliations:** 1 Department of Woodworking Industrial Engineering, Faculty of Technology, Gazi University, 06560 Ankara, Türkiye; ndongel@gazi.edu.tr (N.D.); himirzi@gazi.edu.tr (H.Ö.I.); cevdet@gazi.edu.tr (C.S.); 2 Design and Wood Technology, Faculty of Architecture, University of Applied Sciences, 70000 Ferizaj, Kosovo; agron.bajraktari@ushaf.net

**Keywords:** wood material, lamination, reinforcement, support layer

## Abstract

In this study, the modulus of elasticity and flexural strength properties of laminated wood elements reinforced with steel mesh with different mesh openings were determined. In accordance with the purpose of the study, three- and five-layer laminated elements were produced from scotch pine (*Pinus sylvestris* L.) wood material, which is widely used in the wood construction industry in Türkiye. The 50, 70, and 90 mesh steel used as the support layer was placed between each lamella and pressed with polyvinylacetate (PVAc-D_4_) and polyurethane (PUR-D_4_) adhesives. Afterward, the prepared test samples were kept for 3 weeks at 20 °C temperature and 65 ± 5% relative humidity for 3 weeks. The flexural strength and modulus of elasticity in flexural of the prepared test samples were determined according to the TS EN 408: 2010+A1 standard by the Zwick universal tester. Multiple analysis of variance (MANOVA) was carried out using MSTAT-C 1.2 software to determine the effect of the modulus of elasticity and flexural strength on the obtained flexural properties, the mesh opening of the support layer, and the adhesive type. When the differences within or between groups were significant with a margin of error of 0.05, achievement rankings were made using the Duncan test on the basis of the least significant difference. According to the results of the research, the highest bending strength (120.3 N/mm^2^) was obtained in three-layer samples reinforced with 50 mesh steel wire and bonded with Pol-D4 glue, and the highest modulus of elasticity (8969.3 N/mm^2^) was obtained in three-layer samples reinforced with 50 mesh steel wire and bonded with Pol-D4 glue. As a result, the reinforcement of the laminated wood material with steel wire had an increasing effect on the strength. Accordingly, the use of 50 mesh steel wire can be recommended to increase mechanical properties.

## 1. Introduction

Since the past, furniture has had an important place in terms of possessions because it meets both physical and psychological needs. Wood is an engineering material widely used in interior and exterior decoration applications due to its superior properties, such as its ease of processing, paintability, low energy consumption during processing, availability in various colors and patterns, low permeability of sound and heat [1,2,3,4]. Despite its many advantages, wood also has some disadvantages, such as its hygroscopicity, heterogeneity, and size limitation [5].

Today’s technology has increased the durability of wood materials and paved the way for the production of many new wood materials, such as plywood, particleboard, and other panel products. Wood material has been preferred as a raw material in construction elements for the last 40 years, and although it is used frequently, it is mostly in the form of timber obtained from tree trunks or wood pieces [6]. Especially, evergreen, coniferous, and mature trees are seen as a source of structural timber. As with various other construction materials, wood material is available in different qualities (grades) and has many standardized features and sizes [7].

While the disadvantages such as the heterogeneous structure and limited size possibilities of wood are eliminated, the improvement of the mechanical resistance properties can be achieved with lamination technology (Glulam: Glue-laminated wood) [8]. Layered timber has been used since the 1800s. Research on this material started in the USA in the 1930s in Forest Products Laboratories [9]. Under a prolonged load, wood undergoes a viscoelastic creep, which requires a constant load that varies over time. When the load applied to the reinforced glulam beam changes over time, both the strength and stiffness of the beam decrease [10]. Once pre-stress is applied, the distortion becomes even more significant. Therefore, it is of great theoretical and engineering importance to understand the long-term mechanical performance of reinforced glulam beams and clarify the effect of creep [11].

Reinforcement in glulam beams is a technique that provides greater advantages in both increased stiffness and strength, with structural members having higher mechanical performance [12]. Reinforcement can be achieved using natural fibers or polymeric (artificial) fibers, which are usually bonded internally or externally to the laminate of the stretched region of the beams [13]. It has been observed that reinforcement with metal elements, which can be applied to both the stretched and compressed regions of the glulam parts, is effective in reducing deflection and increasing the loading capacity [14]. Metal has been one of the most widely used materials for reinforcement since the 1960s. Steel bar, steel strip, steel or aluminum sheets, and steel-knitted wire mesh are the best examples. Reinforcing wooden structures with steel materials is both effective and cost-effective [15,16]. Steel-reinforced beams show that the behavior of reinforced beams is completely different from that of non-reinforced ones. The strengthening process changed the failure mode from fragile to durable and increased the load-carrying capacity of the beams [7]. It was determined that for simply reinforced beams, stiffness increased by 25.9%, ultimate load increased by 48.1%, and ductility increased by 43.8%. For reinforced and pre-stressed beams, stiffness increased by 37.9%, ultimate load increased by 40.2%, and ductility increased by 79.1% [14].

In a study that proposed that close-mounted steel rods could be used to reinforce glulam bamboo beams, a total of five glulam bamboo beams, one unreinforced and four reinforced, were constructed and tested to break under a four-point loading system, and the bending behavior was examined by comparing the differences. The experimental results showed that the load-bearing capacity and cross-sectional stiffness of the reinforced beams increased significantly compared with those of the unreinforced beam. It was also found that steel bars mounted close to the surface shared the tensile stress of bamboo beams and worked effectively during the loading process. In addition, the plane section assumption of the cross-sectional stress distribution along the height was verified, and an analytical model was proposed to predict the section stiffness of reinforced bamboo beams [17].

The use of reinforcing mesh on laminated surfaces increased the ultimate load capacities of the tested beams. The highest ultimate load capacities were observed in tests of adhesive-laminated beams, which were reinforced with polyurethane adhesive using steel wire reinforcement nets and produced using five laminated layers in the direction perpendicular to the lamination surface [18].

The results of a study that used one precast concrete, one post-tensioned concrete, one porous steel, and one solid timber in the construction of four one-way parking garages were intriguing. The resulting comparison showed that there was little difference in the energy of the structural systems used for car parks under material best practices. While solid timber was more suitable even in the worst-case scenario, it was observed that it lost its advantageous position against its cement equivalent and high-recycled-content steel [19].

It was shown that the proposed rational reinforcement of wooden beams increased their bearing capacity by 175% and reduced bearing deformation by 85%. The study revealed the high efficiency of the application of the strengthening method in the roof beams and floors of the buildings [20].

Under constant loading conditions, the total stress value of the steel bars decreased by 17.5%, 13.6%, and 9.1%, respectively. The ratio of the long-term deflection of the beam mid-span to the total deflection was 26.9%. As the strengthening ratio increased, the stress loss of the steel bars decreased, and the long-term deflection rate also decreased [11].

As can be seen in the literature summarized above, wood is used for different purposes under different conditions. To achieve high success with smaller sections, wood is subjected to various processes and reinforced with different materials. This study aimed to determine the bending strength and elasticity properties in the bending of glulam beams produced in three and five layers by placing a steel wire mesh with 50, 70, and 90 mesh pore openings between the layers obtained from Scotch pine (*Pinus sylvestris* L.). The results of the study showed that reinforced laminated beams can be used in applications with longer support spans in the wooden structure system compared with solid wood.

## 2. Materials and Methods

### 2.1. Materials

#### 2.1.1. Wood

Scotch pine (*Pinus sylvestris* L.) used in the preparation of the test samples was selected according to criteria such as natural color uniformity, smoothness of fibers, absence of knots, absence of reaction wood, and absence of fungal and insect damage. Test samples were formed into 7 and 4.2 mm lamellas, respectively, according to the 3- and 5-layered state, by a wood saw and planning machines. The wood material, which became lamellae, was stacked and maintained at 20 ± 2 °C and 65 ± 5% relative humidity until the equilibrium moisture content was 12%.

#### 2.1.2. Stainless Steel Wire Mesh

**Figure 1:**
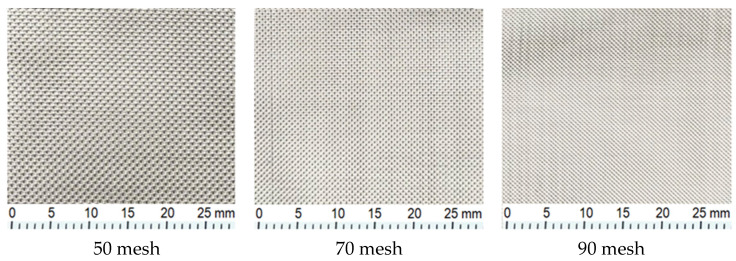
Stainless steel wire mesh samples with different pore openings.

Stainless steel wire meshes are used as braided materials in various places because of the continuity of their mechanical properties as well as their ability to preserve the aesthetic appearance and brightness on their surfaces for a long time and not deform at high temperatures [21]. They are preferred because they have a long life, do not require maintenance, and have high mechanical resistance [22]. In this study, steel wires with 50, 70, and 90 mesh pore openings were used. The wire diameters were 0.18 mm, 0.12 mm, and 0.10 mm, respectively, and the pore spacing was 330 μm, 242 μm, and 180 μm (Figure 1).

#### 2.1.3. Adhesives

Polyvinylacetate (PVAc-D_4_) and polyurethane (PUR-D_4_) adhesives, which are commonly used in the wood industry, were preferred in this study. The recommendations of the manufacturer (Klebreit) were followed for application. The properties of PVAc-D_4_ were as follows: viscosity at 20 °C: 13 ± 2 mPas; color: white; application amount: 120–200 gr/m^2^; open time: 6–10 min. The properties of PUR-D_4_ were a viscosity of 8 ± 1 MPa at 20 °C, yellowish brown color, an application amount of 100–200 gr/m^2^, and an open time of 20–25 min [23].

### 2.2. Preparation of Test Samples

The test samples were prepared with dimensions of 21 mm × 30 mm × 400 mm according to the TS EN 408: 2010+A1 standard [24]. PVAc-D_4_ and PUR-D_4_ were applied to the 7 and 4.2 mm-thick lamellas prepared using air-dried Scots pine (*Pinus sylvestris* L.) and reinforced with 50-, 70-, and 90-opening steel wire mesh reinforcement. and non-reinforced (control) experimental groups were formed.

Two types of specimens were prepared with and without the reinforcement layer. Steels with 3 different mesh properties (50, 70, and 90) were used between each layer of the samples consisting of 3 and 5 lamellas reinforced by the support layer. For each variable, 10 samples were prepared from both the reinforced and non-reinforced experimental groups. While gluing the samples, 180–200 gr/m^2^ adhesive was applied with a brush on both surfaces of the lamellas and pressed under 1.1 N/mm^2^ pressure. After waiting for at least 24 h in the press, the samples were cut with a saw into dimensions of 21 mm × 30 mm × 400 mm.

### 2.3. Conducting of Experiments

**Figure 2:**
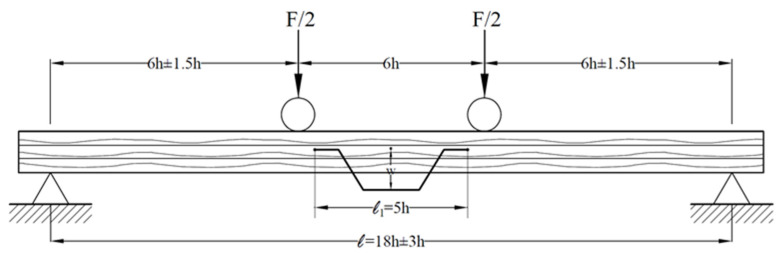
The experimental setup.

The flexural strength and modulus of elasticity in flexural of the prepared test samples were determined according to the TS EN 408: 2010+A1 standard by the Zwick tester [24]. They were determined under a static load from 4 points on the Zwick tester. The experimental setup is given in Figure 2.

(1) $$f_{m}=\frac{3F_{max}l_{2}}{bt^{2}}\;$$

$f_{m}$: Flexural strength (N/mm^2^),

$F_{max}$: Maximum load (N),

$l_{2}$: 16 times the thickness (mm),

*b*: Width (mm),

h: Thickness (mm).

(2) $$E_{m}=\frac{l^{3}\left(F_{2}-F_{1}\right)\;{\left(l-l_{1}\right)}^{3}}{4\left(W_{2}-W_{1}\right)b_{1}\times h_{1}^{3}}$$

$E_{m}$: Modulus of elasticity (N/mm^2^),

l: Length (mm),

$l_{1}$: Distance between supports (mm),

$b_{1}$: Width (mm),

$h_{1}$: Thickness (mm),

*F*_2_ − *F*_1_: Increase in the load ratio on the right part of the load deformation curve (N),

*W*_2_ − *W*_1_: Increase in deformation corresponding to *F*_2_ − *F*_1_, (mm).

**Figure 3:**
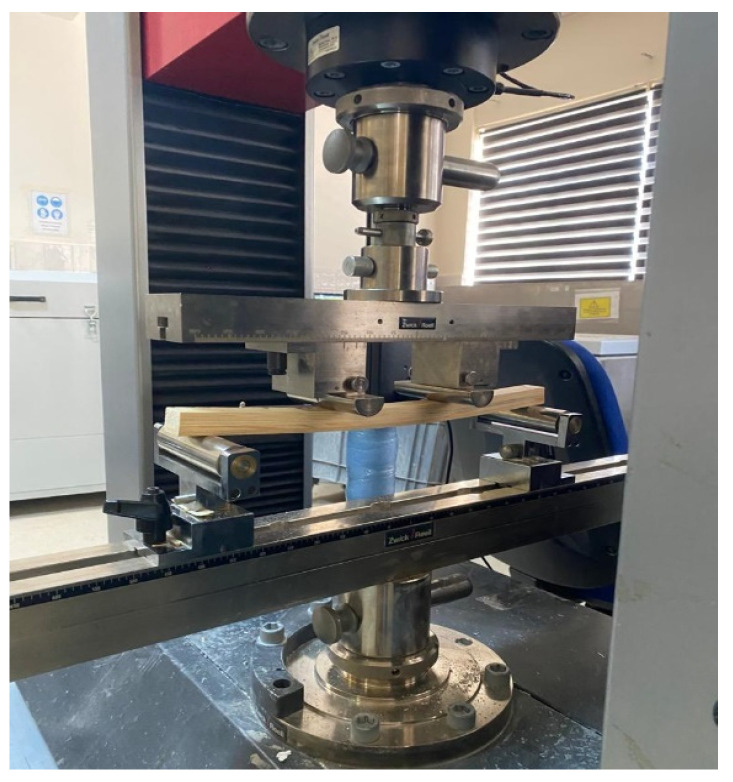
Performing flexural strength and modulus of elasticity.

The 4-point flexural strength ($f_{m})$ and modulus of elasticity $\left(E_{m}\right)$ of the test specimens placed (Figure 3) at a distance of 366 mm between the supports were calculated using Equations (1) and (2) [24].

The proportional comparison of the reinforcement to the flexural strength and flexural modulus of elasticity were calculated according to Equations (3) and (4).

(3) $$P_{fm}=\frac{f_{m_{SW}}-f_{m_{GB}}}{f_{m_{SW}}}\times 100\;\left(\%\right)$$

(4) $$P_{Em}=\frac{E_{m_{SW}}-E_{m_{GB}}}{E_{m_{SW}}}\times 100\;\left(\%\right)$$

$P_{fm}$: Proportional comparison of the flexural strength (%),

$P_{fm}$: Proportional comparison of the modulus of elasticity (%),

$f_{m_{SW}}$: Flexural strength value of solid wood (N/mm^2^),

$f_{m_{GB}}$: Flexural strength value of glulam beam (N/mm^2^).

*SW*: Solid wood,

*GB*: Glulam beam.

### 2.4. Statistical Analysis

Multiple analysis of variance (MANOVA) was carried out using MSTAT-C 1.2 package software to determine the effect of the modulus of elasticity and flexural strength on the obtained flexural properties, the mesh opening of the support layer, and the adhesive type. When the differences within or between groups were significant with a margin of error of 0.05, achievement rankings were made using the Duncan test on the basis of the least significant difference (LSD). As a result, in cases in which the modulus of elasticity and flexural strength properties were important, we attempted to obtain a lamination combination with a high level of success.

## 3. Results and Discussion

### 3.1. Flexural Strength

The statistical values regarding the modulus of elasticity and flexural strength of non-reinforced beams made of 3 layers of lamellas with a thickness of 7 mm and reinforced beams made of 5 layers of 4.2 mm-thick lamellas are given in Table 1.

When the flexural strength values given in Table 2 are examined, it can be seen that there are differences according to the adhesive type, the number of layers, and the characteristics of the reinforcement material. The results of the analysis of variance to determine the factor affecting the flexural strength are given in Table 2.

The differences between groups in terms of the effects of the sources of variance on the flexural strength properties—adhesive type, number of layers, and reinforcement type; adhesive type–number of layers, adhesive type–reinforcement type, and number of layers–pore openings binary interactions; and the adhesive type–number of layers–reinforcement type triple interaction—were statistically significant (*p* ≤ 0.05).

**Figure 4:**
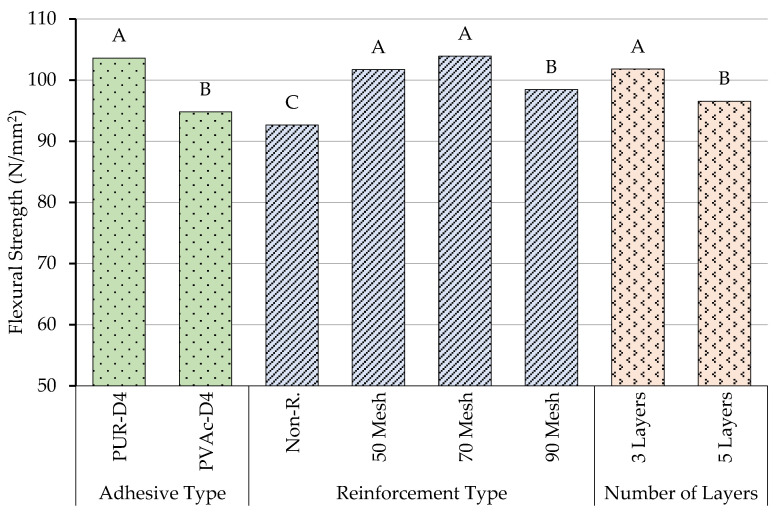
Homogeneity for adhesive type, reinforcement type, and number of layers. A: The highest value of flexural strength, C: The lowest value.

According to the results of the comparative Duncan homogeneity test performed to determine the importance of the adhesive type on flexural strength properties, the highest flexural strength properties were obtained in beams produced with polyurethane glue. Although the production of beams with polyurethane or polyvinylacetate glue showed different effects on flexural strength properties, beams produced with polyvinylacetate glue had the lowest value in terms of flexural strength properties. The homogeneity test results for adhesive type, reinforcement type, and number of layers are shown in Figure 4.

According to the results of the comparative Duncan homogeneity test (LSD ± 2.67) performed to determine the importance of the number of layers to the flexural strength properties, the highest flexural strength (A: 101.8 N/mm^2^) was obtained in beams produced with three layers. Although the production of beams with three layers and five layers showed different effects on flexural strength properties, five-layer beams had the lowest value (B: 96.5 N/mm^2^) in terms of flexural strength properties.

According to homogeneity test results for reinforcement types related to flexural strength, the lowest value (C: 92.64 N/mm^2^) was obtained from beams produced with no reinforcement. Although 50 mesh and 70 mesh showed similar properties in terms of the reinforcement type on flexural strength properties, the highest value (A: 103.9 N/mm^2^) was seen in beams produced using 70 mesh.

**Figure 5:**
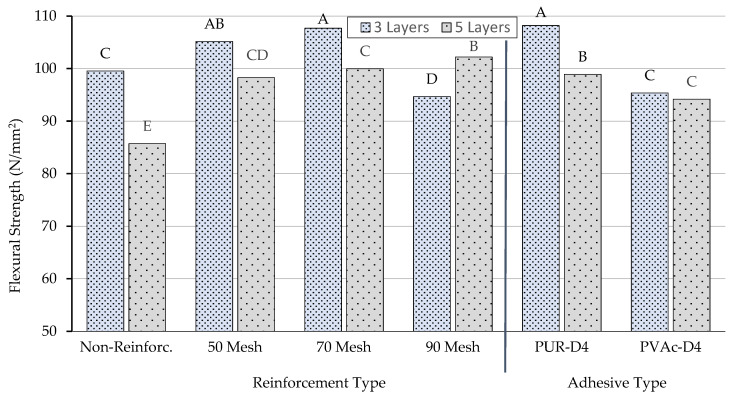
Homogeneity for interaction of reinforcement type–number of layers and adhesive type–number of layers. A: The highest value of flexural strength, E: The lowest value.

The homogeneity test results for the interactions of reinforcement type–number of layers and adhesive type–number of layers are shown in Figure 5.

According to the results of the homogeneity test (LSD ± 3.78) performed to determine the importance of the bilateral interaction between the number of layers and the reinforcement type to the flexural strength properties, the lowest value was obtained for non-reinforced beams produced with five layers, and the highest value was obtained for 70 mesh reinforced beams produced with three layers. In addition, three-layer non-reinforced beams and five-layer 70 mesh reinforced beams showed similar properties.

According to the results of the homogeneity test (LSD ± 2.70) performed to determine the importance of the bilateral interaction between the number of layers and the adhesive type to the flexural strength properties, the lowest value was obtained for polyvinylacetate-glued beams produced with five layers, and the highest value was obtained for polyurethane-glued beams produced with three layers. Beams produced using polyvinylacetate glue showed the same properties for three and five layers.

**Figure 6:**
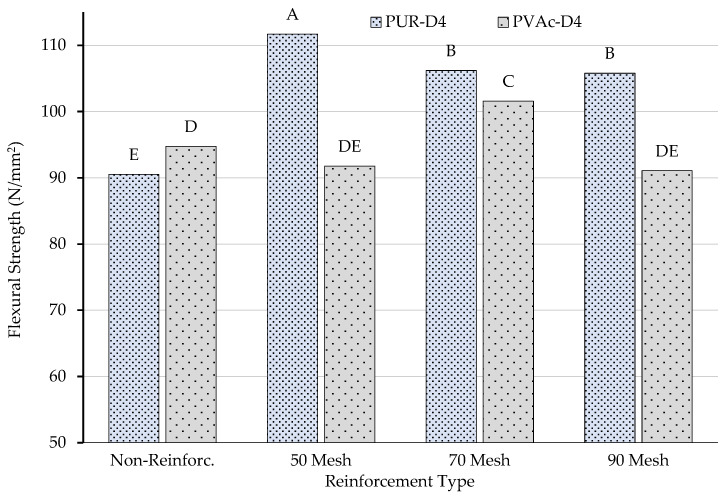
Homogeneity for interaction of reinforcement type–adhesive type. A: The highest value of flexural strength, E: The lowest value.

The Duncan test results for the interaction of reinforcement type–adhesive type are given in Figure 6.

According to the results of the homogeneity test (LSD ± 3.78) performed to determine the importance of the bilateral interaction between the reinforcement type and adhesive type to the flexural strength properties, the lowest value (E: 90.53 N/mm^2^) was obtained for the polyurethane-glued beams produced with no reinforcement, and the highest value (A: 111.7 N/mm^2^) was obtained for the polyurethane-glued beams produced with 50-opening steel mesh wire reinforcement. While beams produced using polyvinylacetate glue showed the same properties in 50-mesh and 90-mesh reinforcements, 70-mesh and 90-mesh reinforcements showed the same properties as beams produced using polyurethane glue.

The triple-interaction Duncan results for adhesive type–number of layers–reinforcement type on flexural strength are given in Table 3.

According to the results of the homogeneity test carried out to determine the importance of the triple interaction of adhesive type–number of layers–pore openings on the flexural strength properties, while the highest flexural strength (120.30 N/mm^2^) was obtained for 50 mesh steel-reinforced beams with polyurethane adhesive, the lowest flexural strength (77.88 N/mm^2^) was obtained for non-reinforced beams with polyurethane adhesive produced with five layers. There was no statistical difference between five-layer glued laminated wood beams bonded with PVAc-D_4_. Additionally, there was no difference between the three-layer non-reinforced material bonded with PUR-D_4_ and the five-layer 50 mesh and 70 mesh reinforcement material (LSD ± 5.34).

### 3.2. Modulus of Elasticity

The results of the analysis of variance to determine the factor affecting the modulus of elasticity are given in Table 4.

The difference between the groups in terms of the effects of the sources of variance on the flexural elasticity modulus—adhesive type, number of layers, reinforcement type, adhesive type–reinforcement type binary interaction, and number of layers–reinforcement type binary interaction—were statistically significant (*p* ≤ 0.05). However, the adhesive type–number of layers binary interaction level was not statistically significant. Due to the anisotropic nature of the wood material, the difference between the fiber length and wood elasticity modulus values significantly affects the measured forces [25,26].

**Figure 7:**
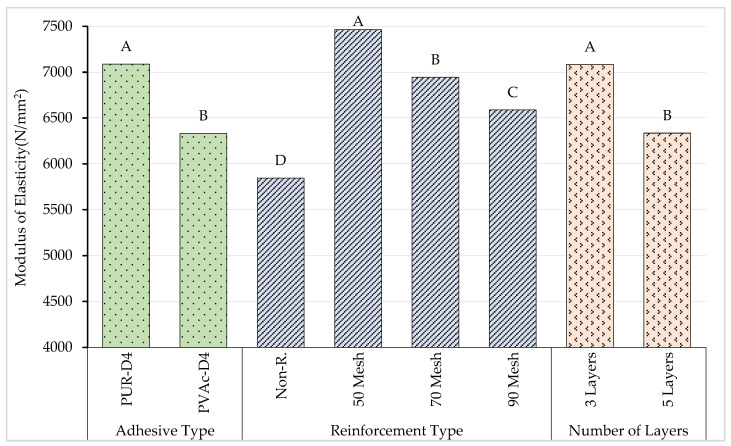
Homogeneity for adhesive type, reinforcement type, and number of layers. A: Higest modulus of elasticity, D: Lowest modulus of elasticity.

According to the results of the comparative Duncan homogeneity test performed to determine the importance of adhesive types to the modulus of elasticity, the highest modulus of elasticity was obtained for beams produced with polyurethane glue. Although the production of beams with polyurethane or polyvinylacetate glue showed different properties on the modulus of elasticity, beams produced with polyvinylacetate glue had the lowest value in terms of the modulus of elasticity. The homogeneity test results for the adhesive type, reinforcement type, and number of layers are shown in Figure 7.

According to the results of the comparative Duncan homogeneity test performed to determine the importance of the number of layers to the modulus of elasticity, the highest modulus of elasticity (A: 7086 N/mm^2^) was obtained for beams produced with three layers. In addition, five-layer beams had the lowest (B: 6336 N/mm^2^) value in terms of the modulus of elasticity.

According to the results obtained, the lowest value was obtained for beams produced with no reinforcement. The highest value (A: 7465 N/mm^2^) in terms of reinforcement types on the elasticity modulus was seen in the beams produced using 50 mesh.

**Figure 8:**
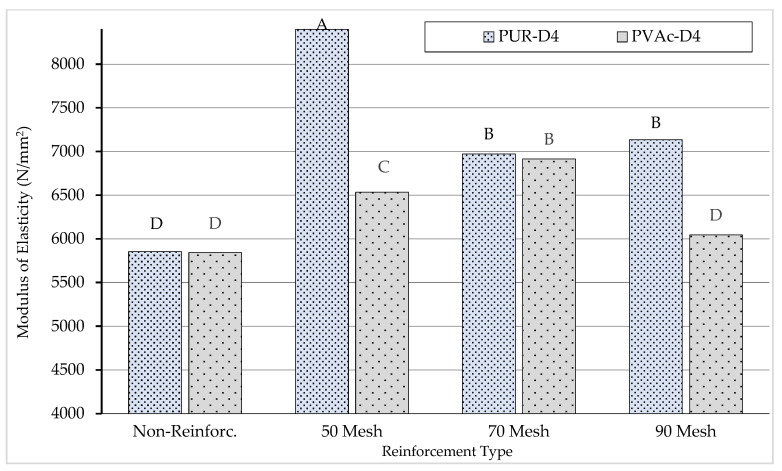
Homogeneity for interaction of adhesive types-reinforcement types. A: Higest modulus of elasticity, D: Lowest modulus of elasticity.

The Duncan homogeneity test performed to determine the importance of the binary interaction of adhesive type–reinforcement type to the modulus of elasticity is shown in Figure 8.

According to the results of the homogeneity test (LSD ± 328.1) carried out to determine the importance of the binary interaction of adhesive type–reinforcement type to the modulus of elasticity, the lowest value (D: 5841 N/mm^2^) was obtained for the polyvinylacetate-glued beams produced with no reinforcement, and the highest value (A: 8398 N/mm^2^) was obtained for the polyurethane-glued beams produced with 50-opening steel mesh wire reinforcement.

**Figure 9:**
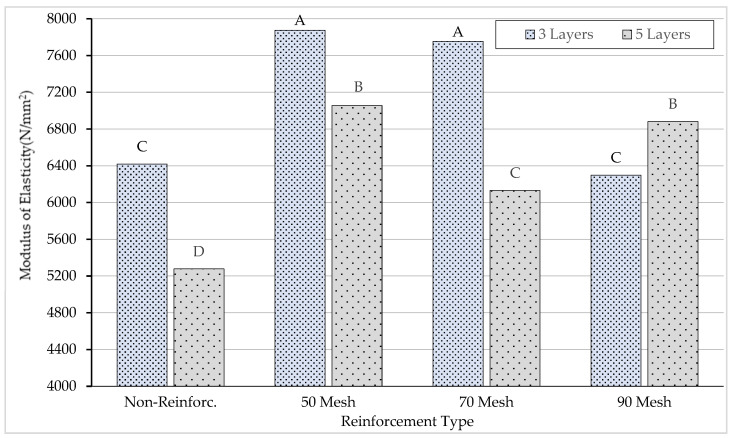
Homogeneity for interaction of the number of layers–reinforcement type. A: Higest modulus of elasticity, D: Lowest modulus of elasticity.

The Duncan homogeneity test performed to determine the importance of the binary interaction of the number of layers–reinforcement type on the modulus of elasticity is given in Figure 9.

According to the results of the homogeneity test (LSD ± 328.1) carried out to determine the importance of the layer number–reinforcement type binary interaction on the modulus of elasticity, the lowest value (D: 5277 N/mm^2^) was obtained for non-reinforced beams produced with five layers, and the highest value (A: 7876 N/mm^2^) was obtained from 50 mesh-reinforced beams produced with three layers.

The triple-interaction Duncan results for adhesive type–number of layers–reinforcement type on the modulus of elasticity are given in Table 5.

**Figure 10:**
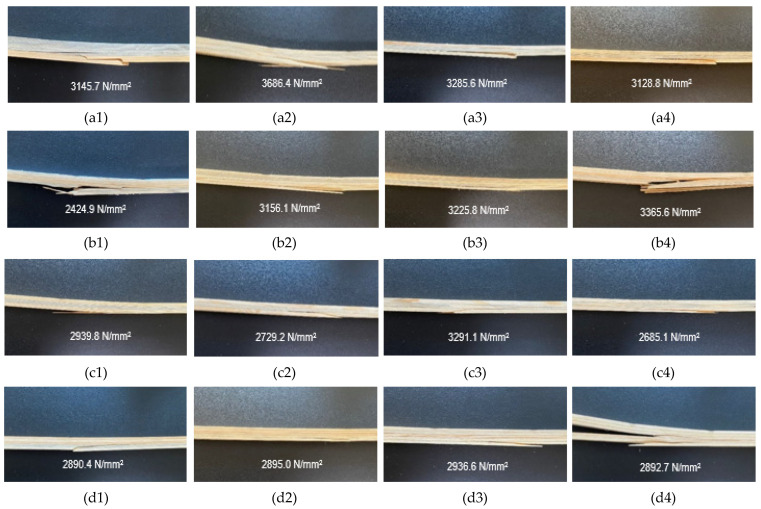
Some of the test samples after experiments. (**a1**) PUR—three layers, (**a2**) PUR—three layers, 50 mesh, (**a3**) PUR—three layers, 70 mesh, (**a4**) PUR—three layers, 90 mesh, (**b1**) PUR—five layers, (**b2**) PUR—5 layers, 50 mesh, (**b3**) PUR—5 layers, 70 mesh, (**b4**) PUR—5 layers, 90 mesh, (**c1**) PVAc—three layers, (**c2**) PVAc—three layers, 50 mesh, (**c3**) PVAc—three layers, 70 mesh, (**c4**) PVAc—three layers, 90 mesh, (**d1**) PVAc—five layers, (**d2**) PVAc—five layers, 50 mesh, (**d3**) PVAc—five layers, 70 mesh, (**d4**) PVAc—five layers, 90 mesh.

According to the results of the homogeneity test (LSD ± 464.0) carried out to determine the importance of the triple interaction of adhesive type–number of layers–reinforcement type to the modulus of elasticity, while the highest flexural strength (8969 N/mm^2^) was obtained for 50 mesh steel-reinforced beams with polyurethane adhesive produced with three layers, the lowest flexural strength (5140 N/mm^2^) was obtained for non-reinforced beams with polyurethane adhesive produced with five layers. The F_max_ values and deformation types of the test samples are given in Figure 10.

The use of steel wire mesh between layers in the production of reinforced beams increased the flexural strength and modulus of elasticity in flexure. As can be seen in Figure 10(b4,d4), in the use of 90 mesh steel-knitted wire, five-layer beams suffered more breakage than three-layer beams, regardless of the adhesive type. Additionally, it should be noted here that the glue line, which has a significant effect on the deformation of the beam between the layers, is damaged during bending [27,28].

The proportional comparison results of the effect of reinforcement on flexural strength and flexural elasticity modulus are given in Table 6.

In terms of the effect on the flexural elasticity modulus, there was an increase of 60% in the reinforcements made with 50 steel mesh and polyurethane adhesive. There was a 31% increase in flexural strength for the same combination.

## 4. Conclusions

The present study aimed to obtain glulam beams that would have high strength properties in terms of performance in the place of use by using steel wire mesh with different pore openings, different types of adhesives, and different numbers of layers. For this purpose, between the layers of glulam beams, 50, 70, and 90 mesh steel wire, which is considered more cost-effective, was used as reinforcement. Additionally, lamella thicknesses of 4.2 mm (for five layers) and 7 mm (for three layers) were produced with the same final thickness as the glulam beams. The flexural strength and modulus of the elasticity properties of the reinforced glulam beams were determined. The obtained data were compared with beams produced with no reinforcement. As a result of the experiment, non-reinforced glulam beams and reinforced glulam beams were evaluated statistically according to adhesive type, number of layers, and pore openings.

In terms of adhesive type, the highest flexural strength value was obtained for polyurethane (PUR-D_4_) glue, and the lowest flexural strength value was obtained for polyvinylacetate (PVAc-D_4_) glue. Moreover, in terms of adhesive type, the highest modulus of elasticity properties was obtained for PUR-D_4_ glue, and the lowest modulus of elasticity properties was obtained for PVAc-D_4_ glue.

In terms of adhesive type, PUR-D_4_ glue had good results for the highest flexural strength and modulus of elasticity, while PVAc-D_4_ glue had low results. This result can be interpreted as follows: polyurethane glue establishes a stronger chemical bond between the lamellae compared to polyvinylacetate glue.

The highest flexural strength and modulus of elasticity values were obtained for three layers, and the lowest flexural strength value was obtained for five layers.

In terms of pore openings, the highest flexural strength value was obtained for 50 mesh, and the lowest flexural strength value was obtained for non-reinforced glulam beams. Again, in terms of pore openings, the highest modulus of elasticity properties was obtained for 50 mesh, and the lowest modulus of elasticity was obtained for non-reinforced glulam beams. In line with the data obtained, it can be interpreted that as the pore opening increases, the flexural strength decreases. Here, it can be interpreted that porous reinforcement materials may be preferred over plate-shaped reinforcement material for use between layers. Simultaneously, the use of 90 mesh can be recommended in cases in which elasticity is desired, while the use of 50 mesh can be recommended in applications that require rigidity.

To conclude, it is predicted that satisfying results can be obtained by diversifying adhesives with different wood species in the construction sector and by experimenting with different reinforcements and various numbers of layers and sequences. Additionally, a preliminary idea was formulated that materials with a porous structure, as reinforcement, could further increase the healing effect.

In the experiments, delamination was observed in some of the samples. This indicated that the adhesive adhesion was not sufficient. With the use of porous support layers, in addition to adhesion, it can have a further healing effect thanks to mechanical bonding.

The experimental results showed that reinforcement improved the bending strength of the glulam beams. The most important reason for this is that the strength properties of the steel material were higher than the wood material, and if they are used together, this feature has a positive effect on the strength of the wood material.

## Figures and Tables

**Table 1:** Mean and coefficient of variation values of the flexural strength and modulus of elasticity (N/mm^2^).

Adhesive Type	Number of Layers	Reinforcement Type	Number of Samples	Flexural Strength (N/mm^2^)		Modulus of Elasticity (N/mm^2^)	
				Mean	COV	Mean	COV
PUR-D_4_	3	Non-reinforcement	10	103.2	4.6	6568.5	9.5
		50 Mesh	10	120.3	2.4	8969.3	6.7
		70 Mesh	10	107.6	1.7	7465.6	8.2
		90 Mesh	10	101.7	5.8	6757.7	9.5
	5	Non-reinforcement	10	79.6	11.3	5139.8	6.8
		50 Mesh	10	103.1	5.2	7825.8	9.3
		70 Mesh	10	104.7	1.9	6480.7	8.9
		90 Mesh	10	110.0	6.3	7510.2	5.4
PVAc-D_4_	3	Non-reinforcement	10	96.0	2.8	6267.6	10.1
		50 Mesh	10	90.1	8.0	6781.9	6.2
		70 Mesh	10	107.9	7.4	8044.7	6.1
		90 Mesh	10	87.7	4.9	5835.4	5.4
	5	Non-reinforcement	10	93.6	6.0	5413.6	6.6
		50 Mesh	10	93.5	4.9	6283.8	4.0
		70 Mesh	10	95.3	3.1	5783.8	13.1
		90 Mesh	10	94.5	4.5	6254.4	3.4

**Table 2:** Analysis of variance results for flexural strength.

Source of Variance	Degrees of Freedom	Sum of Squares	Mean Square	F Value	*p* ≤ 0.05
Adhesive Type (A)	1	3220.230	3220.230	113.238	0.0000 *
Number of Layers (B)	1	1014.049	1014.049	35.659	0.0000 *
Interaction (AB)	1	588.289	588.289	20.687	0.0000 *
Reinforcement Type (C)	3	2652.834	884.278	31.095	0.0000 *
Interaction (AC)	3	3260.366	1086.789	38.217	0.0000 *
Interaction (BC)	3	2320.004	773.335	27.194	0.0000 *
Interaction (ABC)	3	1833.480	611.160	21.491	0.0000 *
Error	144	4095.016	28.438		
Total	159	18984.268			

*: The difference is significant at a level of 0.05.

**Table 3:** Triple-interaction Duncan results of adhesive type–number of layers–reinforcement type on flexural strength (N/mm^2^).

Reinforcement Type	PUR-D_4_				PVAc-D_4_			
	3 Layers		5 Layers		3 Layers		5 Layers	
	$\bar{x}$	HG	$\bar{x}$	HG	$\bar{x}$	HG	$\bar{x}$	HG
Non-Reinforcement	103.20	CD	77.88	H **	95.96	E	93.56	EF
50 Mesh	120.30	A *	103.10	CD	90.08	FG	93.45	EF
70 Mesh	107.60	BC	104.70	CD	107.90	BC	95.28	EF
90 Mesh	101.70	D	110.00	B	87.67	G	94.48	EF
LSD ± 5.339								

$\bar{x}$: Arithmetic mean. HG: Homogeneity group. *: The highest flexural strength. **: The lowest flexural strength.

**Table 4:** Analysis of variance results for modulus of elasticity.

Source of Variance	Degrees of Freedom	Sum of Squares	Mean Square	F Value	*p* ≤ 0.05
Adhesive Types (A)	1	22893580.550	22893580.550	82.9539	0.0000 *
Number of Layers (B)	1	22488826.095	22488826.095	81.4872	0.0000 *
Interaction (AB)	1	94775.169	94775.169	0.3434	NS
Reinforcement Type (C)	3	55344914.544	18448304.848	66.8466	0.0000 *
Interaction (AC)	3	23773747.689	7924582.563	28.7144	0.0000 *
Interaction (BC)	3	27042864.738	9014288.246	32.6629	0.0000 *
Interaction (ABC)	3	6120967.578	2040322.526	7.3930	0.0001 *
Error	144	39741076.618	275979.699		
Total	159	197500752.981			

*: The difference is significant at the level of 0.05.

**Table 5:** Homogeneity for interaction of adhesive type–number of layers–reinforcement type on modulus of elasticity (N/mm^2^).

**Reinforcement Types**	PUR-D_4_				PVAc-D_4_			
	3 Layers		5 Layers		3 Layers		5 Layers	
	$\bar{x}$	HG	$\bar{x}$	HG	$\bar{x}$	HG	$\bar{x}$	HG
Non-Reinforcement	6568	D	5140	G **	6268	DE	5414	FG
50 Mesh	8969	A *	7826	BC	6782	D	6284	DE
70 Mesh	7466	C	6481	D	8045	B	5784	EF
90 Mesh	6758	D	7510	C	5835	EF	6254	DE
LSD ± 464.0								

$\bar{x}$: Arithmetic mean. HG: Homogeneity group. *: The highest MOE. **: The lowest MOE

**Table 6:** Proportional comparison of reinforcement effect (%).

Number of Layer	Reinforcement	Flexural Modulus of Elasticity (N/mm^2^)					Flexural Strength (N/mm^2^)				
		Solid Wood (N/mm^2^)	PUR-D_4_		PVAc-D_4_		Solid Wood (N/mm^2^)	PUR-D_4_		PVAc-D_4_	
			$E_{m}$ (N/mm^2^)	Change (%)	$E_{m}$ (N/mm^2^)	Change (%)		$f_{m}$ (N/mm^2^)	Change (%)	$f_{m}$ (N/mm^2^)	Change (%)
3 Layers	Non-Reinforc.	5587.9	6568.5	17.6	6267.6	12.2	93.9	103.2	9.9	95.9	2.2
	50 Mesh		8969.3	60.5	6781.9	21.4		120.3	28.1	90.1	−3.1
	70 Mesh		7465.6	33.6	8044.7	33.6		107.6	14.6	107.9	15.0
	90 Mesh		6757.7	20.9	5835.4	8.2		101.7	8.3	87.7	−6.3
5 Layers	Non-Reinforc.		5139.8	−8.0	5413.6	−3.1		77.9	−17.0	93.6	−0.4
	50 Mesh		7825.8	40.0	6283.8	12.5		103.1	9.8	93.5	−0.5
	70 Mesh		6480.7	16.0	5783.8	15.9		104.7	11.5	95.1	1.5
	90 Mesh		7510.2	34.4	6254.4	34.4		110.0	17.15	94.5	0.6

## Data Availability

The raw/processed data required to reproduce these findings cannot be shared at this time, as the data also form part of an ongoing study.
